# Drug Stability Analysis by Raman Spectroscopy

**DOI:** 10.3390/pharmaceutics6040651

**Published:** 2014-12-22

**Authors:** Chetan Shende, Wayne Smith, Carl Brouillette, Stuart Farquharson

**Affiliations:** Real-Time Analyzers, Inc., Middletown, CT 06457, USA; E-Mails: chetan@rta.biz (C.S.); wayne@rta.biz (W.S.); carl@rta.biz (C.B.)

**Keywords:** drug stability analysis, drug degradation, Raman spectroscopy, multivariate analysis, astronaut health

## Abstract

Pharmaceutical drugs are available to astronauts to help them overcome the deleterious effects of weightlessness, sickness and injuries. Unfortunately, recent studies have shown that some of the drugs currently used may degrade more rapidly in space, losing their potency before their expiration dates. To complicate matters, the degradation products of some drugs can be toxic. Here, we present a preliminary investigation of the ability of Raman spectroscopy to quantify mixtures of four drugs; acetaminophen, azithromycin, epinephrine, and lidocaine, with their primary degradation products. The Raman spectra for the mixtures were replicated by adding the pure spectra of the drug and its degradant to determine the relative percent contributions using classical least squares. This multivariate approach allowed determining concentrations in ~10 min with a limit of detection of ~4% of the degradant. These results suggest that a Raman analyzer could be used to assess drug potency, nondestructively, at the time of use to ensure crewmember safety.

## 1. Introduction

Astronauts suffer from a number of maladies caused by long-term weightlessness and radiation exposure, such as space motion sickness (SMS), cephalad fluid shifts, sleep deprivation, reduced immune response, and loss of bone and muscle mass [1,2,3,4,5,6]. To counter these effects, astronauts have available medicines to either treat the physiological changes or the symptoms. In the case of the International Space Station (ISS), a medical kit is included to aid the health of the astronauts. The kit contains pharmaceutical drugs that range from acetaminophen (Tylenol^®^) for headaches and pain to promethazine for SMS to epinephrine for allergic reactions and cardiac arrest. However, the size limitations imposed by the ISS (or future space craft) restrict the number of drug types, as well as their quantity. Furthermore, the value of the drugs is limited to the period that they maintain 90% of their potency, *i.e.*, their shelf-lives, which are typically 1 to 2 years. The active pharmaceutical ingredient (API) will degrade over time, most often due to heat and moisture, which promote hydrolysis of the API. As expected, the ISS drugs are replaced according to their listed shelf-lives or more specifically, the expiration dates. However, it was recently shown that the rate of degradation for some drugs may accelerate in space [7,8], possibly due to radiation. It was reported that some of the drugs fell below 90% potency before their expiration date. To complicate matters, the degradation products of some drugs can be toxic, such as *p*-aminophenol formed from acetaminophen, which can cause liver damage [9].

Consequently, there is a need for an analyzer to measure both the API concentration and its degradation products in spaceflights. Such an analyzer would have immediate value in its ability to assess the potency of a drug at the time of use to ensure crewmember safety. It would also have long-term value in its ability to perform degradation studies aboard the ISS to better define the shelf-lives of drugs in that environment.

Drug manufacturers employ standard stress tests to establish shelf-life and identify degradation products, as outlined by the International Conference on Harmonisation (ICH) [10]. These tests accelerate environmental storage conditions, such as hydrolysis, photolysis, oxidation, and heat. Once the degradation products are formed, they are identified by a number of analytical techniques, such as infrared spectroscopy (IR), Raman spectroscopy, nuclear magnetic resonance spectroscopy (NMR), and/or mass spectrometry (MS). Once identified, a method is developed to quantify the degradation products. Most often the method is high performance liquid chromatography (HPLC) [11]. Numerous HPLC methods for various pharmaceuticals, such as acetaminophen, have been published [12,13,14,15,16,17,18]. Unfortunately, HPLC is not suited for the proposed application, as it is relatively labor intensive, it is time consuming (tens of minutes), and it requires consumables (solvents and columns). Samples must be dissolved in a carrier solvent and filtered prior to injection into the column, and must often be pretreated to remove interferents, such as the inactive ingredients (excipients). Furthermore, different carrier solvents, columns and conditions are typically required for each drug class.

While IR, NMR, and MS can in principal be used to quantify API degradation, each has a serious limitation with regard to this application. Just like HPLC, sample pretreatment, using consumables, is required for NMR and MS. In addition, these analyzers are large and heavy. While mid-IR and near-IR have been successfully used to confirm the identity of drugs and drug formulations using the reflection mode [19,20,21], the transmission mode is required to perform quantitative analysis. The latter mode, in general, requires diluting the sample in a non-absorbing solvent, which would preclude administering the drug sample [22].

Raman spectroscopy measures the vibrational modes of a sample. A spectrum consists of a wavelength distribution of peaks corresponding to molecular vibrations specific to the sample being analyzed. Chemicals, such as drugs, can be identified by the frequency and quantified by the intensity of the peaks. In the last 20 years, considerable technological advances, such as stable diode lasers, sharp wavelength transition optical filters, and high quantum efficiency detectors, have made Raman spectroscopy standard equipment in analytical laboratories, and more recently, allowed the development of lightweight, portable systems [23]. An attractive advantage to this technique is that samples do not have to be extracted or prepared, and the analysis is non-destructive. A laser is simply focused into the sample to generate the Raman radiation, which is collected by the spectrometer for analysis.

Recently, Raman spectroscopy has been successfully used to verify contents of drugs within their packaging [22], measure the composition and uniformity of drug pills [24,25,26,27,28,29], identify street drugs [30,31], and determine drug authenticity [32]. Based on these successes, we present a preliminary investigation of the ability of Raman spectroscopy to quantify mixtures of four drugs; acetaminophen, azithromycin, epinephrine, and lidocaine, with their primary degradation products. Here we limit the analysis to each API and its primary degradant to demonstrate the basis of this approach.

## 2. Experimental Section

Acetaminophen (paracetamol, Tylenol^®^) and *p*-aminophenol were purchased from US Pharmacopeia (Rockville, MD, USA) at a purity of >99%. Azithromycin (Zithromax^®^), azaerythromycin A, lidocaine (xylocaine or lignocaine), 2,6-dimethylaniline, epinephrine (adrenaline, adrenalin), and nor-epinephrine (noradrenaline) were purchased from Sigma Aldrich (Allentown, PA, USA) at a lower purity of >97.5% (Analytical grade) due to the high cost. The APIs and degradants were ground by mortar and pestle into fine powders to minimize particle size effects [33,34]. Samples were prepared by weighing (Metler Toledo) and adding 1, 4, 9, 19, and 49 mg of the API to 1 mg of the degradant to produce 50%, 20%, 10%, 5%, and 2% mass percent mixtures, respectively. This concentration series was chosen to cover a wide range with a focus on the low concentrations germane to the described application. After each addition, the samples were vortex mixed (Scientific Industries, Inc., Bohemia, NY, USA) and again ground by mortar and pestle to improve homogeneity.

All Raman measurements were performed using an FT-Raman spectrometer (RamanID-1064, RTA, Middletown, CT, USA). The spectrometer employed a 1064 nm diode laser (Innovative Photonic Solutions, Monmouth Junction, NJ, USA) that provided 500 mW at the sample, a proprietary interferometer to separate the Raman signal into its component wavelengths, and a single element InGaAs detector (Judson Tech, Montgomeryville, PA, USA). Measurements were performed using 8 cm^−1^ resolution. Samples were placed in a 2 mL vial, mounted on the XY stage above the source laser and measured. Measurements of the pure samples consisted of five one-minute spectra averaged together, each spectrum consisting of 100 scans. Measurements of mixtures consisted of ten one-minute spectra averaged together, obtained at 10 spots, ~300 µm in diameter, spaced 1 mm apart along the length of the vial to compensate for potential mixture inhomogeneities. The average spectrum of the ten spots was used for calculations to better represent the prepared concentrations.

## 3. Results and Discussion

**Figure 1:**
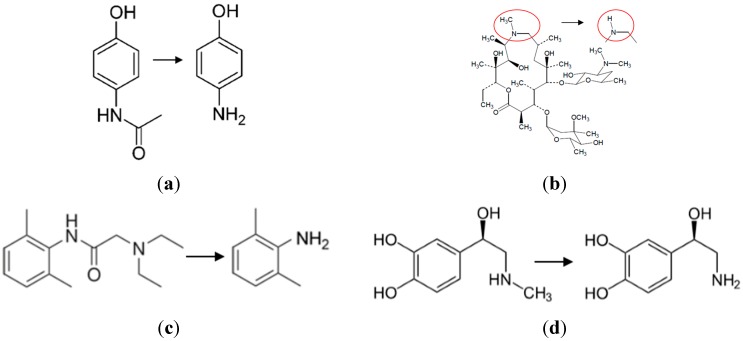
The chemical structures of the four drugs and their primary degradation products. (**a**) acetaminophen to *p*-aminophenol; (**b**) azithromycin to azaerythromycin A; (**c**) lidocaine to 2,6-dimethylaniline; and (**d**) epinephrine to nor-epinephrine.

Four drugs representative of the medications used by NASA astronauts were selected for study: acetaminophen, azithromycin, lidocaine, and epinephrine (Figure 1). Acetaminophen is used primarily as a pain reliever and a fever reducer; azithromycin is used as an antibiotic used for treating middle ear infections, strep throat, and pneumonia; lidocaine is used for relieving skin itching, burning and pain, as well as for minor surgery; and epinephrine is used to treat allergic reactions and stimulate the heart during cardiac arrest.

**Figure 2:**
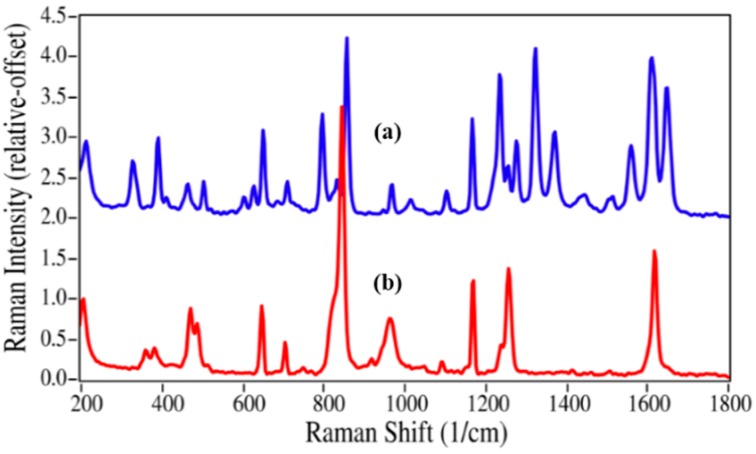
Raman spectra of (**a**) acetaminophen and (**b**) *p*-aminophenol. Spectral conditions: 500 mW at 1064 nm, 8 cm^−1^ resolution, 5 min acquisition (100 scans).

**Figure 3:**
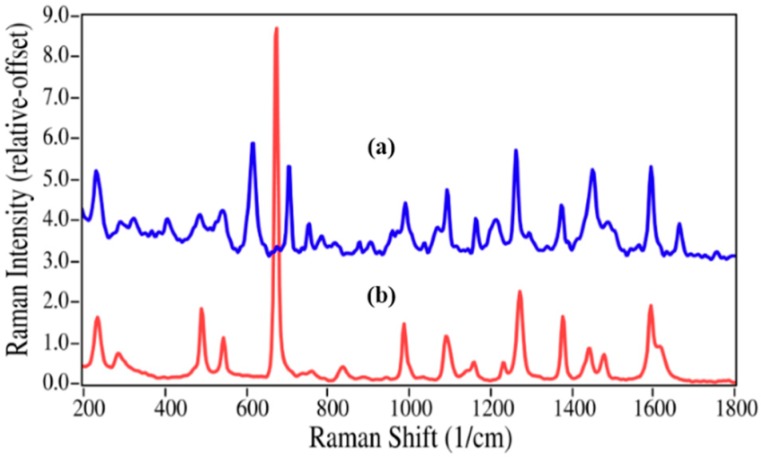
(**a**) Raman spectra of (**a**) Lidocaine (intensity × 5); and (**b**) 2,6-dimethylaniline. Spectral conditions as in Figure 2.

The structures for acetaminophen and lidocaine and their primary degradation products are fairly different and consequently produced Raman spectra with significant differences that could be used to quantify mixtures (Figure 2 and Figure 3). The Raman spectrum of acetaminophen is dominated by peaks at 797, 858, 1236, 1324, 1560, 1611, and 1649 cm^−1^, which are assigned to CNC ring stretching, ring breathing, C–C ring stretching, amide III, amide II, ring stretching, and amide I modes, respectively [35,36,37]. Upon loss of the amide functional group during degradation, the amide bands and the CNC stretching mode disappear, while the increased molecular symmetry results in more intense ring modes for *p*-aminophenol. The Raman spectrum of lidocaine is also dominated by the CNC ring, ring, and amide modes at 617, 706 and 1596, and 1666 cm^−1^, respectively. The same spectral changes are observed for the degradation of lidocaine to 2,6-dimethylaniline, *i.e.*, the 617 and 1666 cm^−1^ peaks disappear, while the ring breathing mode gains intensity and shifts to a lower frequency, *viz.* 706 to 675 cm^−1^.

**Figure 4:**
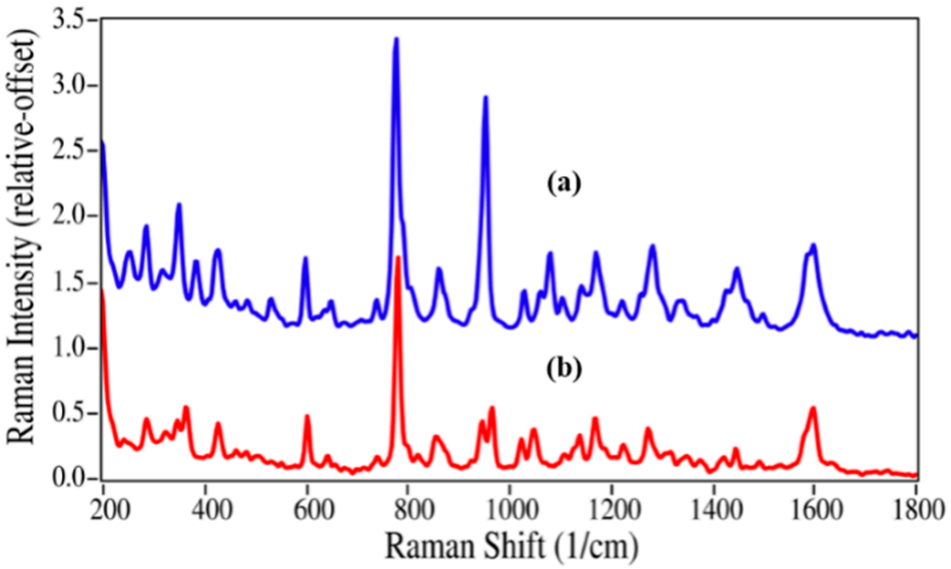
Raman spectra of (**a**) epinephrine and (**b**) nor-epinephrine. Spectral conditions as in Figure 2.

Epinephrine and azithromycin degradation involves replacing a methyl group by a hydrogen atom, and consequently the spectral changes are less substantial. Virtually all of the peaks in the epinephrine Raman spectrum are present in the nor-epinephrine spectrum, which include those at 599, 777, 1081, 1172, 1283 and 1599 cm^−1^ that are assigned to CC=O stretching, ring breathing, CCH bending, CO stretching, CO asymmetric stretching, and aromatic CC stretching modes (Figure 4) [35,38]. However, the unassigned intense peak at 954 cm^−1^ is replaced by a doublet with modest intensity at 947 and 966 cm^−1^, suggesting that this epinephrine peak contains some CNC character.

**Figure 5:**
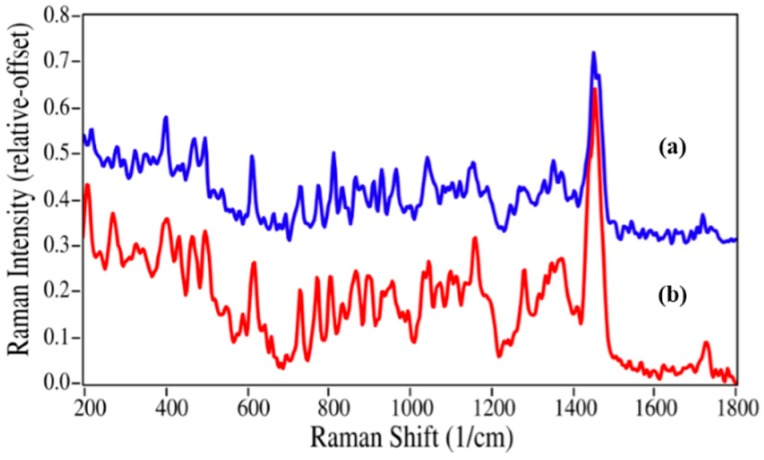
Raman spectra of (**a**) azithromycin and (**b**) azaerythromycin A. Spectral conditions as in Figure 2.

Since azithromycin has numerous methyl groups, the replacement of one group at the nitrogen position of the oxo-6-azacyclopentadecyl ring by a hydrogen atom, as expected, produced only minor changes in the Raman spectrum (Figure 5). The Raman spectrum is dominated by a peak at 1454 cm^−1^ due to the ether stretching modes, while a number of CC and CNC stretching, and CH bending modes appear between 600 and 1200 cm^−1^ [35]. Within this region minor changes do occur; in particular, the peak at 814 cm^−1^ loses intensity and shifts to 805 cm^−1^. While the spectral differences between epinephrine and nor-epinephrine may be sufficient to quantify a mixture, it is clear that the differences between azithromycin and azaerythromycin A may not.

**Figure 6:**
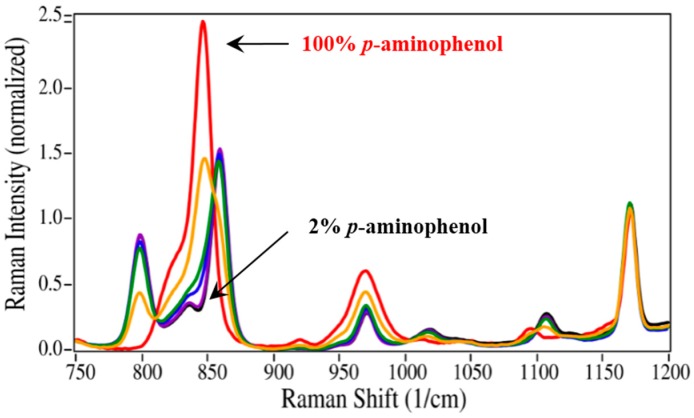
Raman spectra of *p*-aminophenol mixed with acetaminophen at 100, 50, 20, 10, 5, and 2 mass %, all normalized to the 1170 cm^−1^ peak height set equal to 1. Inset: graph and values of prepared *versus* calculated mass % based on peak height at 846 cm^−1^. Spectral conditions as in Figure 2, except ten one-minute spectra were averaged for each concentration.

**Figure 7:**
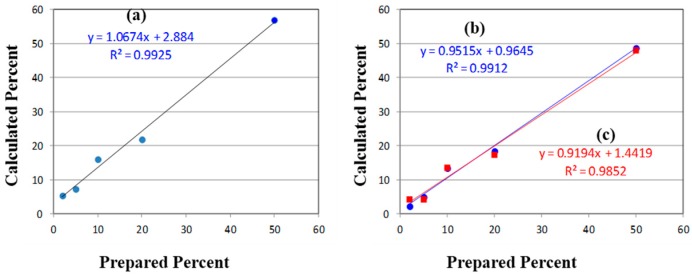
(**a**) Plots of Prepared *versus* Calculated percentages of *p*-aminophenol mixed with acetaminophen determined using (**a**) the Raman peak height at 846 cm^−1^; and the 550–1800 cm^−1^ Raman spectral region using (**b**) raw spectra (circles), and (**c**) first derivatives of the spectra (squares). Ideally, the slope should equal 1.0 and the intercepts 0.0.

The initial quantification of the mixtures employed the traditional method of measuring Raman peak intensities (height or area). It is worth noting that the absolute concentration of the degradant is not necessary, but only its concentration relative to the API. As shown in Figure 2, the best peak for this purpose is the intense ring breathing mode at 846 cm^−1^ for *p*-aminophenol. Since this peak overlaps with the same mode in the acetaminophen spectrum (Figure 6), the peak height was used. The peak height at this frequency for the measured mixtures was scaled to 100% for pure *p*-aminophenol and 0% for pure acetaminophen (Table 1). While the correlation coefficient (*R*^2^) for a plot of the prepared *versus* calculated percentages was close to 1.0, all of the predicted concentrations were high with an intercept of 2.9% (Figure 7). The latter can be taken as the background level, which multiplied by 3 represents a limit of detection (LOD) of 8.7% [39]. The lack of accuracy in the data, represented by the root mean squared error (RMSE), can be attributed to the overlapping of the peaks. It is clear from these data, that traditional peak height analysis will not allow accurate determination of acetaminophen in a medication that has degraded by 10% or less.

Since the univariate approach to quantitation clearly has limitations, a simple multivariate approach was investigated. The approach fit the entire spectrum with classical least squares weighted contributions from the two pure spectra of the drug and its degradant at each wavelength [40]. A software program was written that allows selecting the spectral region to fit, smoothing the spectra, and/or taking the first derivative. Judicious selection of the spectral region allows limiting the calculation to the spectral features that best represent each component in the mixture. Smoothing the spectra minimizes errors associated with fitting the noise instead of the signal. The first derivative minimizes the effects of baseline offset, slope and fluorescence contributions. For the acetaminophen/*p*-aminophenol mixtures, the 550 to 1800 cm^−1^ spectral region of the raw spectra and their first derivatives were used to calculate each sample concentration (Table 1). Both spectral types yielded more accurate concentrations than the simple peak height calculation. More importantly, the spectra-based calculated percentages proved very accurate at the low concentrations, e.g., 4.9% and 2.2% for 5.0% and 2.0%, respectively. The straight line fit to the spectra also yielded a *y*-intercept of 0.96, representing an improvement of a factor of three in the LOD to 2.88%. Smoothing was not used in these calculations, since the spectra contained little noise. In addition, using the first derivative of the spectra was probably not necessary since the spectral baseline was flat. In fact, the first derivative yielded slightly inferior results compared with the raw spectra.

**Table 1:** Prepared and Calculated percentages of *p*-aminophenol mixed with acetaminophen determined using Raman peak height at 846 cm^−1^ and spectral weighting of the spectra and their first derivatives for the 550 to 1800 cm^−1^ region.

% *p*-Aminophenol			
**Prepared**	**Calculated**	**Calculated (550–1800)**	
%	Peak Hit	Spectra	1st der
50	56.8	48.7	48.0
20	21.8	18.4	17.3
10	16.0	13.4	13.5
5	7.3	4.9	4.2
2	5.3	2.2	4.2
*R*^2^	0.993	0.991	0.985
RMSE	4.52	1.78	2.41
Intercept	2.90	0.96	1.44
LOD	8.70	2.88	4.32

The spectral fitting approach was next applied to the remaining drugs and their degradants to determine how well it could calculate low concentrations. The data are summarized in Table 2.

**Table 2:** Prepared and calculated percentages of degradants mixed with their corresponding drug determined using Raman spectra. Spectral regions are indicated.

	Azaerythromycin A	Norepinephrine	2,6-Dimethylaniline
Prepared	Calculated	Calculated	Calculated
%	565–1220	575–1560	450–1050
50	41.3	43.6	48.1
20	22.1	24.6	20.6
10	11.3	8.4	10.4
5	4.3	2.9	9.7 *
2	0.8	0.2	7.3 *
*R*^2^	0.97	0.96	0.99
RMSE	4.09	3.80	1.17
Intercept	1.55	0.045	1.40
LOD	4.65	0.135	4.20

* Not included in the *R*^2^, RMSE, Intercept and LOD calculations.

**Figure 8:**
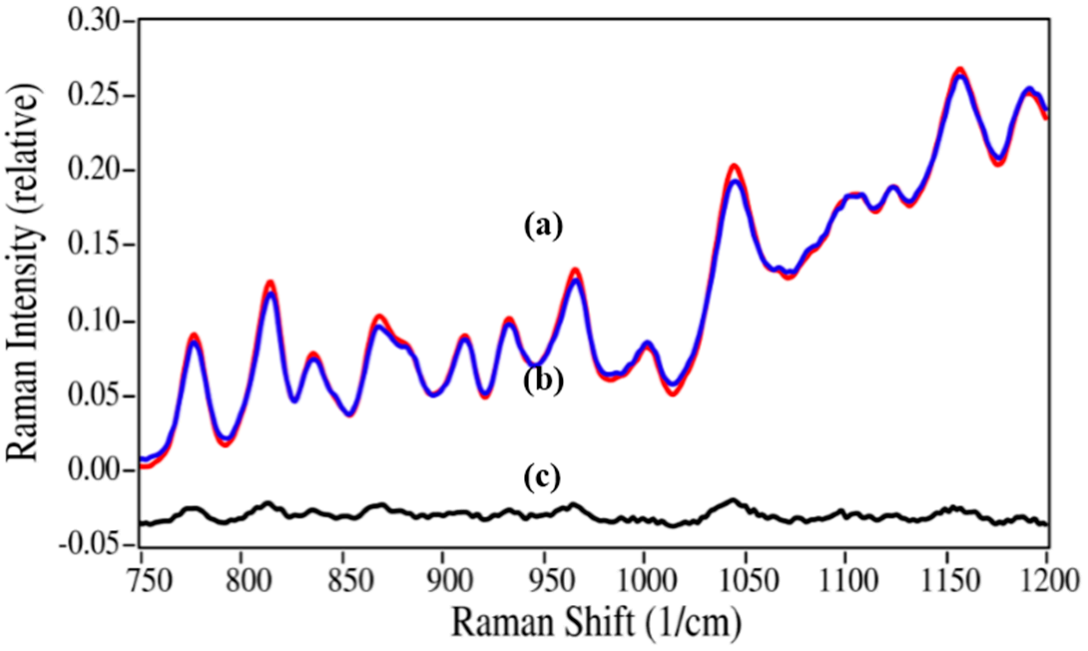
Raman spectrum of (**a**) the 5%/95% azaerythromycin A/azithromycin mixture (red) fit with (**b**) a spectrum created using 4.3% azaerythromycin A and 95.7% azithromycin of their pure spectra; (**c**) The residual of the fit (**a–b**). Spectral conditions as in Figure 6, except 565 to 1220 cm^−1^ region used.

The azithromycin/azaerythromycin A mixtures presented the greatest challenge of the four drugs to the ability of Raman spectroscopy to calculate percentages using spectral weighting, since the spectral differences are very minor (see Figure 5) and the Raman spectra are relatively weak (the *y*-axes of all spectra in Figure 2, Figure 3, Figure 4 and Figure 5 use the same relative scale). Nevertheless, very good results were obtained, as long as the spectral region used to calculate the concentrations was confined to the region where these spectral differences are most apparent. A very good fit to the 5%/95% azaerythromycin A/azithromycin mixture was obtained using 4.3%/95.7% and the 565 to 1220 cm^−1^ region (Figure 8).

While the norepinephrine/epinephrine pair had greater spectral differences than the azaerythromycin A/ azithromycin pair, the accuracies of the calculated values were not quite as good at the low concentrations (Table 2). This may be attributed to the inhomogeneities in the samples and the limited number of spectral measurement points, which may not have adequately represented the concentration for this sample. The lidocaine/2,6-dimethylaniline mixtures presented a different challenge, primarily in terms of sample preparation. Lidocaine is a powder, while 2,6-dimethylaniline is a liquid. The samples were prepared as weight/weight percentages, but at 2,6-dimethylaniline concentrations below 10%, lidocaine did not dissolve, but instead separated. Consequently, Raman spectral measurements were of heterogeneous samples. This created greater error in 2% and 5% 2,6-dimethylaniline concentrations, which were consequently not used in the *R*^2^ and LOD calculations.

## 4. Conclusions

This preliminary investigation demonstrates that Raman spectroscopy has great potential to determine the extent of degradation of active pharmaceutical ingredients nondestructively and without sample preparation in 10 min or less. The average limit of detection of 3.9%, based on *y*-intercepts for the four drugs measured, suggests that 10% degradation can be determined with this ~4% accuracy (RMSE), provided that the tablets, powders, gels or pastes contain a significant percentage of API by mass. Fortunately, many formulations, such as Tylenol^®^ and PLIVA^®^ (azithromycin) are composed of greater than 50% API, viz.: 325 and 250 mg, as part of 400 and 450 mg tablets, respectively [41]. It is also worth noting that, with the exception of titanium dioxide, most excipients generate much weaker Raman signals than APIs [42]. Furthermore, the maximum mass of the degradant in any mixture measured here was 1 mg, indicating that the absolute degradant mass should not limit sensitivity. Nevertheless, future work will expand analysis to actual products containing excipients and explore methods to improve the limits of detection for all of the ISS drugs with a goal of 10% ± 1% degradant. These methods will include various Raman excitation wavelengths, laser powers, more sensitive detectors, acquisition times, optical arrangements (such as transmission [26,43]), and multivariate analysis.
